# Supplementary material for: Coordinated neural progenitor adaptations contribute to primate neocortical downscaling
Source: Sci Adv. 2026 Sep 23;12(39):eaeg5563. doi: 10.1126/sciadv.aeg5563 (PMC13600215; doi:10.1126/sciadv.aeg5563)
Supplement: Supplementary file 1 [file sciadv.aeg5563_sm.pdf]

Science Advances
AAAS

Supplementary Materials for

Coordinated neural progenitor adaptations contribute to primate neocortical downscaling

Lidiia Tynianskaia *et al.*

Corresponding author: Michael Heide, heide.michael@ mh-hannover.de

Sci. Adv. **12**, eaeg5563 (2026)
DOI: 10.1126/sciadv.aeg5563

The PDF file includes:

Figs. S1 to S9
Legends for tables S1 to S3 and S5 to S7
Table S4

Other Supplementary Material for this manuscript includes the following:

Tables S1 to S3 and S5 to S7

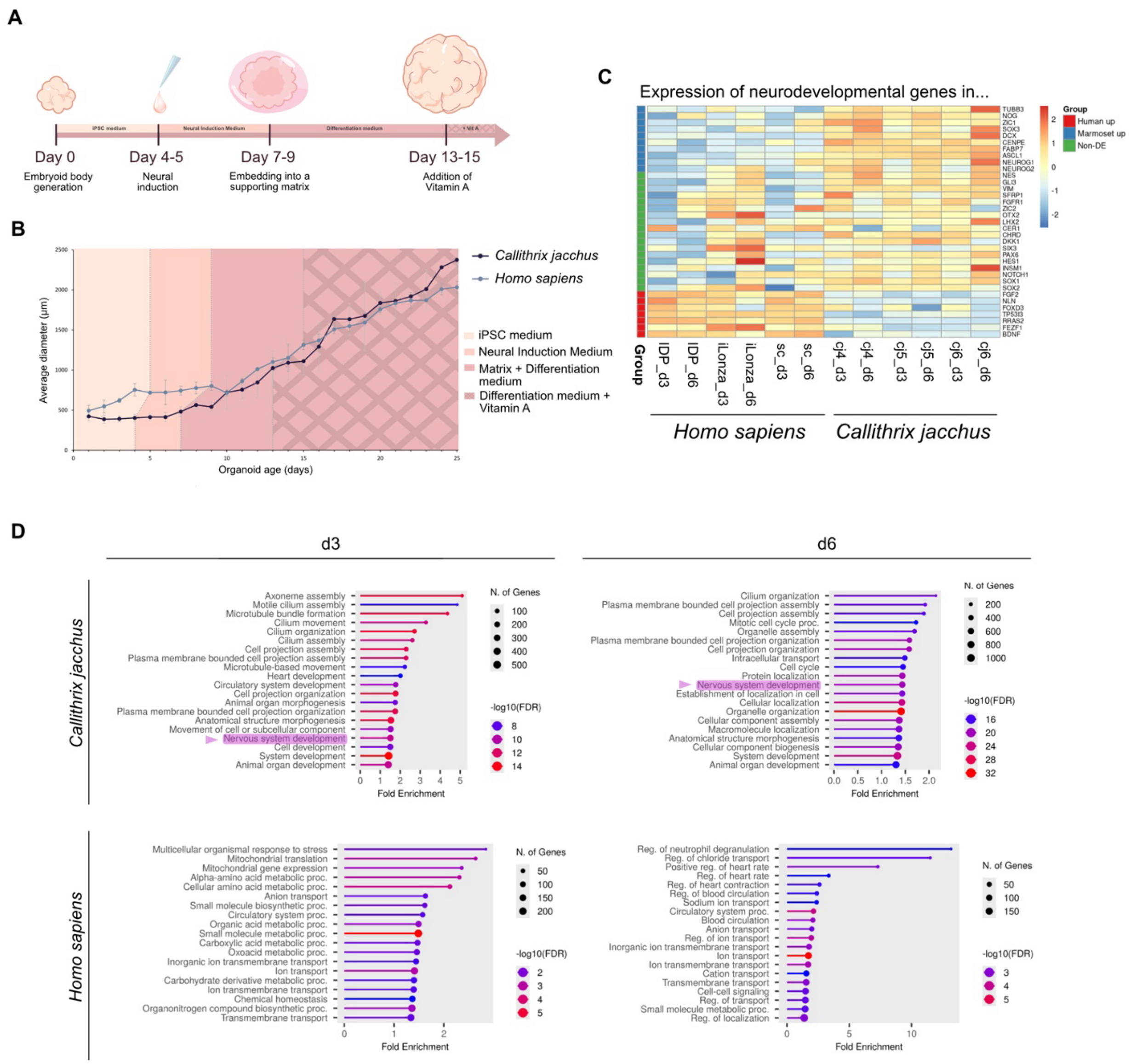

Fig. S1: Marmoset embryoid bodies display smaller overall size but accelerated neural fate acquisition compared to human.

(A) Schematic illustration of the timeline of cerebral organoid generation. **(B)** Growth curve showing the diameter of human and marmoset cerebral organoids analyzed over a 25-day period. Data represent the mean of 1–3 independent batches per time point, each comprising from 7 to 21 cerebral organoids (7 organoids per iPSC line) generated from 3 independent iPSC lines per species (marmoset or human). Error bars, ±SD. **(C)** Heatmap depicting differential gene expression

of neurodevelopmental genes between human (left) and marmoset (right) embryoid bodies (EBs) at day 3 and day 6, based on three biological replicates (cell lines) per condition (species × stage). Genes are grouped by expression pattern: upregulated in human (red), upregulated in marmoset (blue), or non-differentially expressed (green). Differentially expressed genes were defined as those with $|\log_2 FC| > 1$ and $p < 0.01$. **(D)** Gene Ontology (GO): Biological Process enrichment analysis of genes overexpressed in marmoset (top) and human (bottom) EBs at day 3 (left) and day 6 (right).

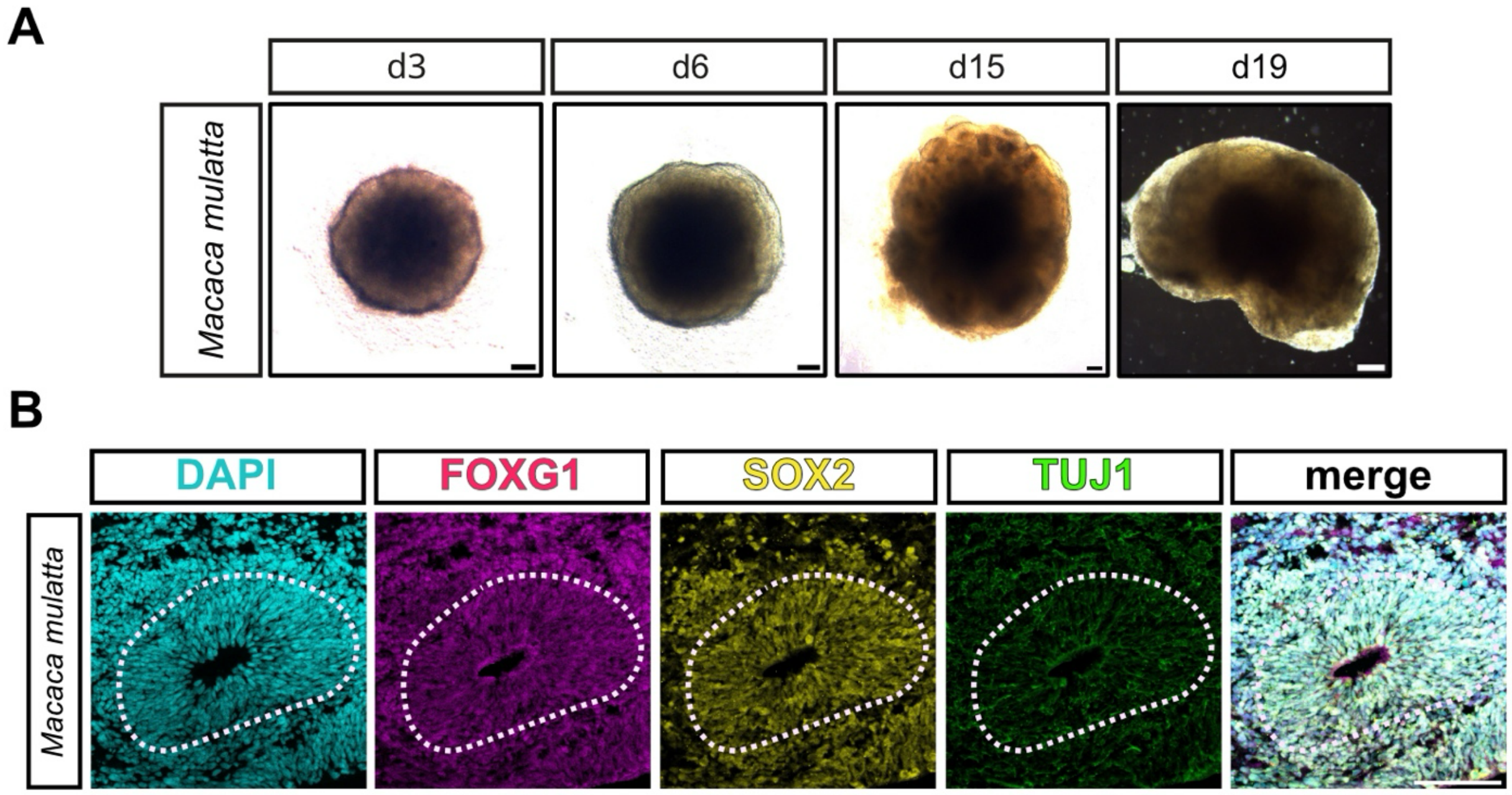

Fig. S2: Rhesus macaque cerebral organoids acquire forebrain identity and express typical markers of ventricle-like structures.

(**A**) Bright field images of rhesus macaque embryoid bodies (3 and 6 days old) and cerebral organoids (15 and 19 days old). Scale bars, 100 µm (d3, d6 and d15), 200 µm (d19). (**B**) Triple immunofluorescence for FOXG1 (magenta), SOX2 (yellow) and TUJ1 (green), combined with DAPI staining (cyan), of 30-day-old rhesus macaque cerebral organoids. Dashed line indicates VZ/basal region border. Scale bars, 100 µm.

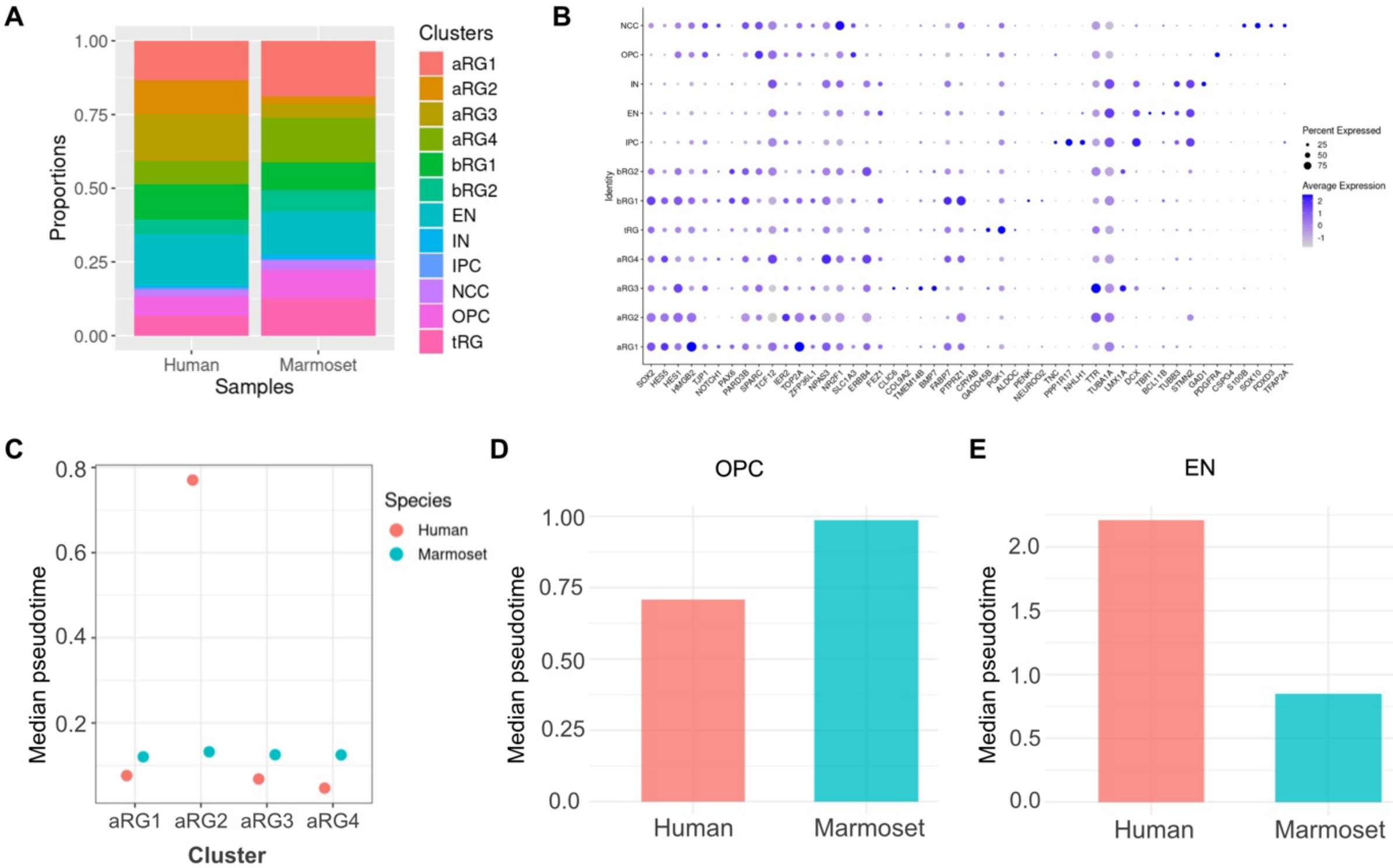

Fig. S3: Marmoset and human 30-day-old cerebral organoids share cell type composition but differ in developmental maturation of aRG and terminal cell types.

(A) Relative contributions of the various cell type clusters to the total cell count in human and marmoset cerebral organoids at day 30. aRG: apical radial glia, tRG: truncated radial glia, bRG: basal radial glia, IPC: intermediate progenitor cell, EN: excitatory neuron, IN: interneuron, OPC: oligodendrocyte progenitor cell, NCC: neural crest cell. **(B)** Dot plot of SCTransform-normalized scRNA-seq expression data showing average expression levels (color) and the percentage of cells expressing selected marker genes as indicated (dot size) in 30-day-old human and marmoset cerebral organoids. **(C–E)** Median pseudotime of 30-day-old human and marmoset cerebral organoids calculated using the Monocle3 standard pipeline, with all cell types rooted to aRG1 (see Methods). Pseudotime distributions were non-normal (Shapiro–Wilk test, $P < 0.05$). Only cross-

species comparisons of median pseudotime for aRGs **(C)**, oligodendrocyte precursors (OPCs; **D**), and excitatory neurons (ENs; **E**) are shown.

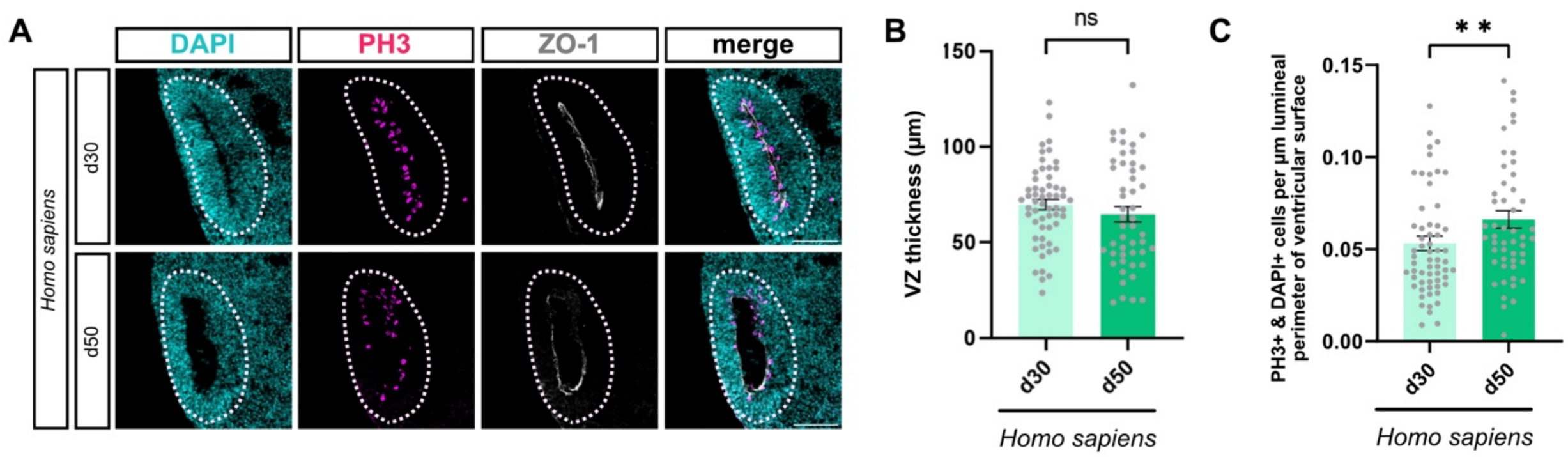

Fig. S4: Human cerebral organoids maintain VZ thickness and consistent aRG mitotic activity during early neurogenesis.

(A) Double immunofluorescence for PH3 (magenta) and ZO-1 (gray), combined with DAPI staining (cyan), in 30- (upper row) and 50 (lower row)-day-old human cerebral organoids. Dashed lines indicate VZ/basal region border. Scale bars, 100 µm. **(B)** Quantification of VZ thickness (µm) measured at four locations spaced 90° apart around a ventricle-like structure in 30- and 50-day-old human cerebral organoids. Data represent the mean of 57 ventricle-like structures from 16 human 30-day-old cerebral organoids, and 49 ventricle-like structures from 12 human 50-day-old cerebral organoids, generated from 3 independent human iPSC lines. Error bars, ±SEM; ns not significant: P=0,2714 (Mann-Whitney test). **(C)** Quantification of PH3+ mitotic cells at the ventricular surface, normalized to the luminal perimeter, in 30- and 50-day-old cerebral organoids. Data represents the mean of 60 ventricle-like structures from 16 human 30-day-old cerebral organoids, and 50 ventricle-like structures from 12 human 50-day-old cerebral organoids, generated from 3 independent human iPSC lines. Error bars, ±SEM; **P=0.0093 (Mann-Whitney test).

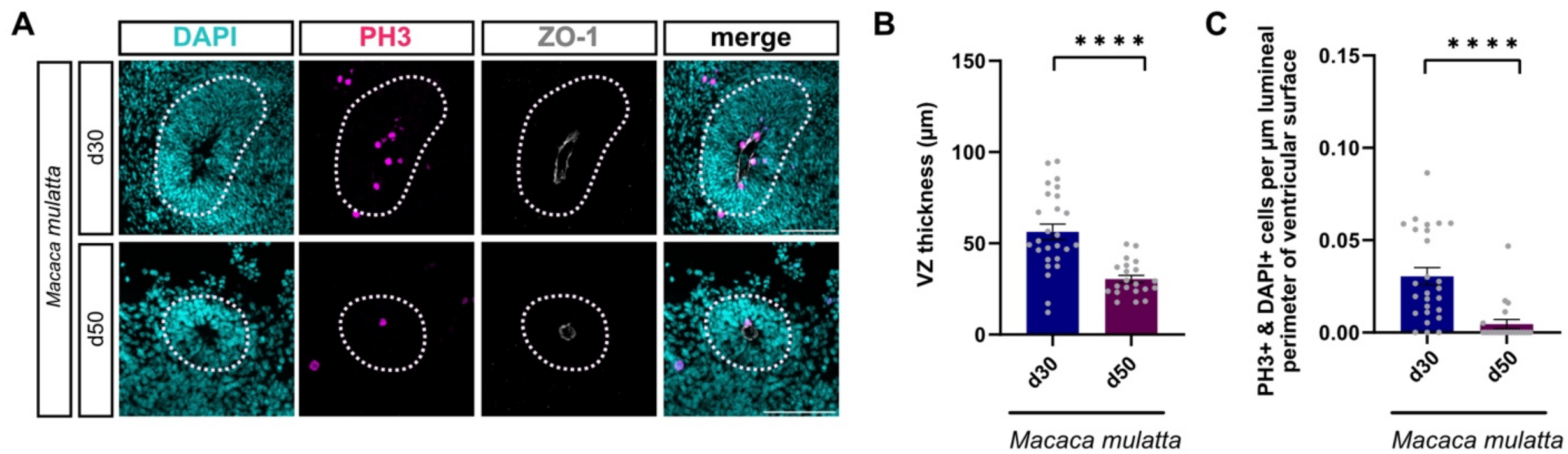

Fig. S5: Rhesus macaque cerebral organoids show a reduction in VZ thickness and aRG mitotic activity between culture day 30 and 50.

(A) Double immunofluorescence for PH3 (magenta) and ZO-1 (gray), combined with DAPI staining (cyan), in 30- (upper row) and 50 (lower row)-day-old rhesus macaque cerebral organoids. Dashed lines indicate VZ/basal region border. Scale bars, 100 µm. **(B)** Quantification of VZ thickness (µm) measured at four locations spaced 90° apart around a ventricle-like structure in 30- and 50-day-old rhesus macaque cerebral organoids. Data represent the mean of 26 ventricle-like structures from 7 rhesus macaque 30-day-old cerebral organoids, and 21 ventricle-like structures from 7 rhesus macaque 50-day-old cerebral organoids, generated from 1 rhesus macaque iPSC line. Error bars, ±SEM; ****P<0,0001 (unpaired t-test). **(C)** Quantification of PH3+ mitotic cells at the ventricular surface, normalized to the luminal perimeter, in 30- and 50-day-old cerebral organoids. Data represents the mean of 26 ventricle-like structures from 7 rhesus macaque 30-day-old cerebral organoids, and 21 ventricle-like structures from 7 rhesus macaque 50-day-old cerebral organoids, generated from 1 rhesus macaque iPSC line. Error bars, ±SEM; ****P<0,0001 (Mann-Whitney test).

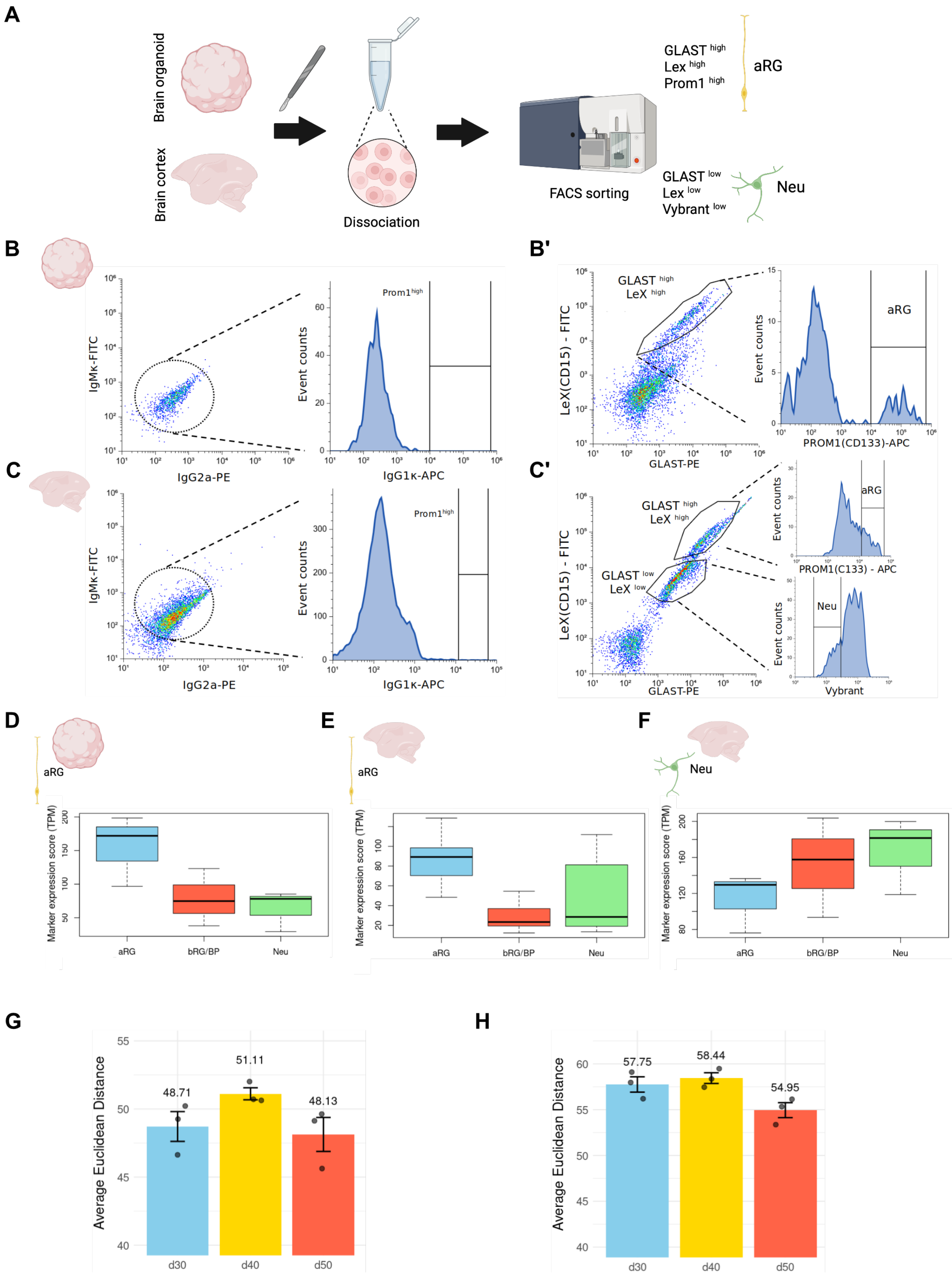
A
Brain organoid
Brain cortex
Dissociation
FACS sorting
GLAST high
Lex high
Prom1 high
aRG
GLAST low
Lex low
Vybrant low
Neu
B
B'
C
C'
IgMκ-FITC
IgG2a-PE
Event counts
IgG1κ-APC
Prom1 high
GLAST high
LeX high
GLAST low
LeX low
LeX(CD15) - FITC
GLAST-PE
PROM1(CD133)-APC
PROM1(C133) - APC
Vybrant
D
E
F
Marker expression score (TPM)
aRG
bRG/BP
Neu
G
H
Average Euclidean Distance
48.71
51.11
48.13
57.75
58.44
54.95
d30
d40
d50

Fig. S6: FACS-enriched aRG from marmoset cerebral organoid show greater transcriptomic similarity to *in vivo* telencephalic aRG than to telencephalic neurons.

(A) Schematic illustration of fluorescence-activated cell sorting (FACS) experimental design used to enrich aRG and neuronal populations from marmoset cerebral organoids (days 30, 40 and 50) and GD90 fetal marmoset telencephalic tissue. **(B, B')** FACS gating strategy to enrich aRG from day 30, 40 and 50 marmoset cerebral organoids using GLAST, LeX, and PROM1 antibodies. Gates were set using isotype control antibodies **(B)**. Apical radial glia were enriched based on high GLAST and LeX signals (left plot), and high PROM1 signal (right plot) **(B')**. **(C, C')** FACS gating strategy to enrich aRG and neurons from GD90 fetal marmoset telencephalic tissue using the same antibodies as in **(B, B')** and Vybrant DNA staining to quantify DNA content. Gates were set using isotype control antibodies **(C)**. Apical radial glia were enriched as described in **(B')**. Neurons were enriched based on low GLAST and LeX signals (left plot), and low Vybrant signal (right plot) corresponding to low DNA content indicative of the G1/G0 phase **(C′).** FACS sorting gates for GLAST, LeX, PROM1, and Vybrant staining in GD90 telencephalon to isolate aRGs and neurons. **(D-F**) Marker gene expression (in transcripts per million, TPM) of key aRG, bRG/bIP, and neuronal markers in FACS-sorted populations of marmoset cerebral organoid aRG. Expression is shown for three cell lines at days 30, 40, and 50 of organoid culture (n = 9). **(D)**, GD90 telencephalic tissue aRG (n = 3) **(E)**, and GD90 telencephalic tissue neurons (n = 3) **(F)**. **(G,H)** Average Euclidean distance between FACS-sorted aRG from 30- (blue), 40- (yellow) and 50 (red)-days-old marmoset cerebral organoids and aRG **(G)** or neurons **(H)** from GD90 fetal marmoset telencephalic tissue, based on expression data of 728 neurodevelopmental genes (see Table S5). The distance was measured from each indicated stage average to every individual GD90

telencephalon (n=3 per time point). Created in BioRender. Heide, M. (2026) https://BioRender.com/0revyu4.

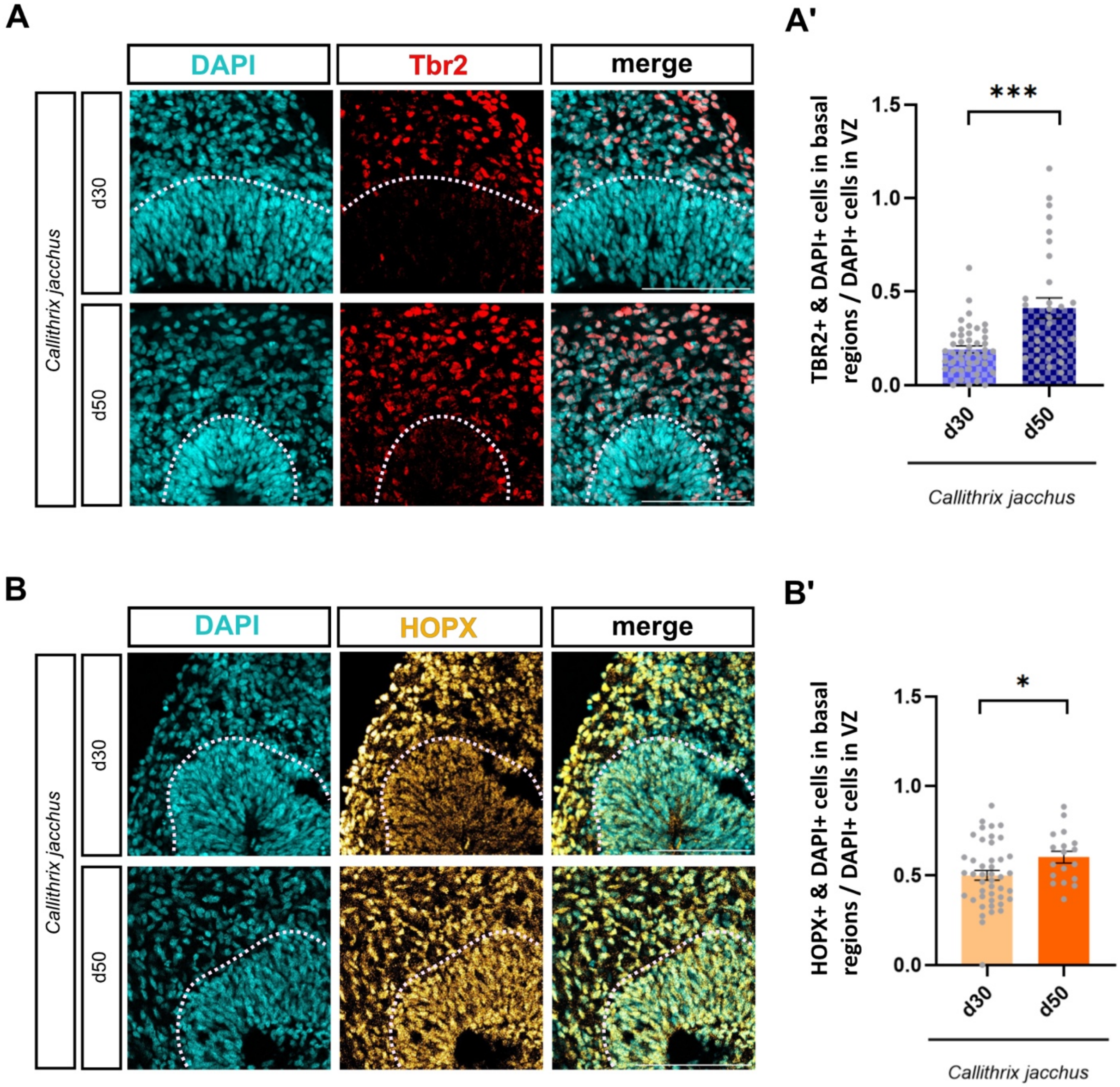

Fig. S7: Marmoset AP delamination leads to increased abundance of BP cell types in cerebral organoids.

(A) Immunofluorescence for Tbr2 (red), combined with DAPI staining (cyan) in 30- (upper row) and 50 (lower row)-day-old marmoset cerebral organoids. Dashed lines indicate VZ/basal region border. Scale bars, 100 µm. **(A')** Quantification of Tbr2+/DAPI+ double-positive cells in the basal region relative to DAPI+ VZ progenitors in the adjacent VZ region of 30- and 50-day-old

marmoset cerebral organoids. Data represent the mean of 44 radial units from 19 marmoset 30-day-old cerebral organoids, and 31 radial units from 23 marmoset 50-day-old cerebral organoids, generated from 3 independent marmoset iPSC lines. Error bars, ±SEM; ***P=0.0004 (Mann-Whitney test). **(B)** Immunofluorescence for HOPX (orange), combined with DAPI staining (cyan), in 30- (upper row) and 50 (lower row)-day-old marmoset cerebral organoids. Dashed lines indicate VZ/basal region border. Scale bars, 100 µm. **(B')** Quantification of HOPX+/DAPI+ double-positive cells in the basal region compared to DAPI+ VZ progenitors in the adjacent VZ region of 30- and 50-day-old marmoset cerebral organoids. Data represent the mean of 44 ventricle-like structures from 15 marmoset 30-day-old cerebral organoids, and 18 ventricle-like structures from 10 marmoset 50-day-old cerebral organoids, generated from 3 independent marmoset iPSC lines. Error bars, ±SEM; *P=0.0377 (unpaired t-test).

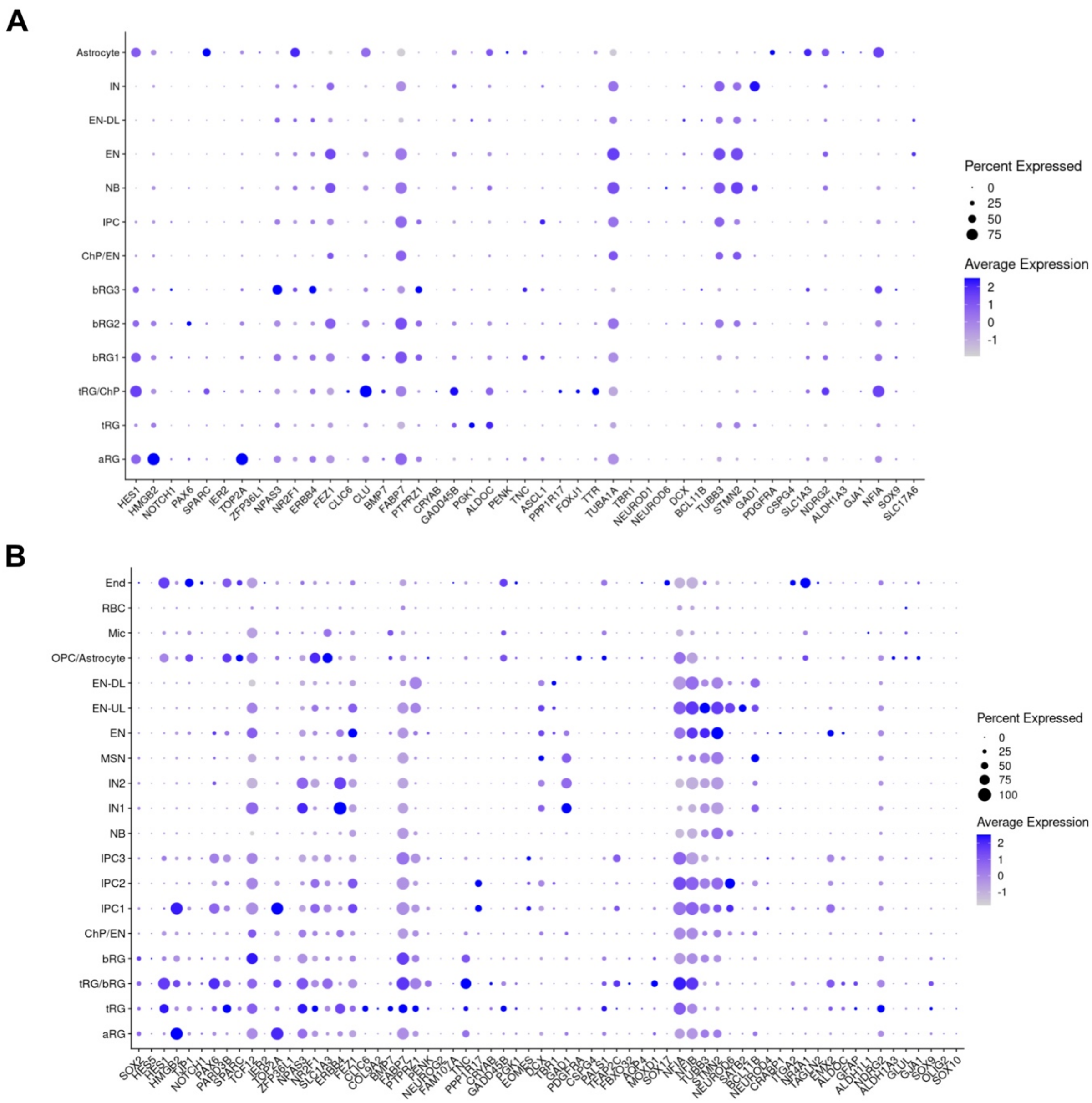

Fig. S8: Marker gene expression confirms the identity of annotated cell types in scRNA-seq data from marmoset day 50 cerebral organoids and GD90 telencephalic tissue.

(A-B) Dot plots of SCTransform-normalized scRNA-seq expression data showing average expression levels (color) and the percentage of cells expressing selected marker genes as indicated (dot size) in day 50 marmoset cerebral organoids **(A)** and GD90 fetal marmoset telencephalic tissue **(B)**.

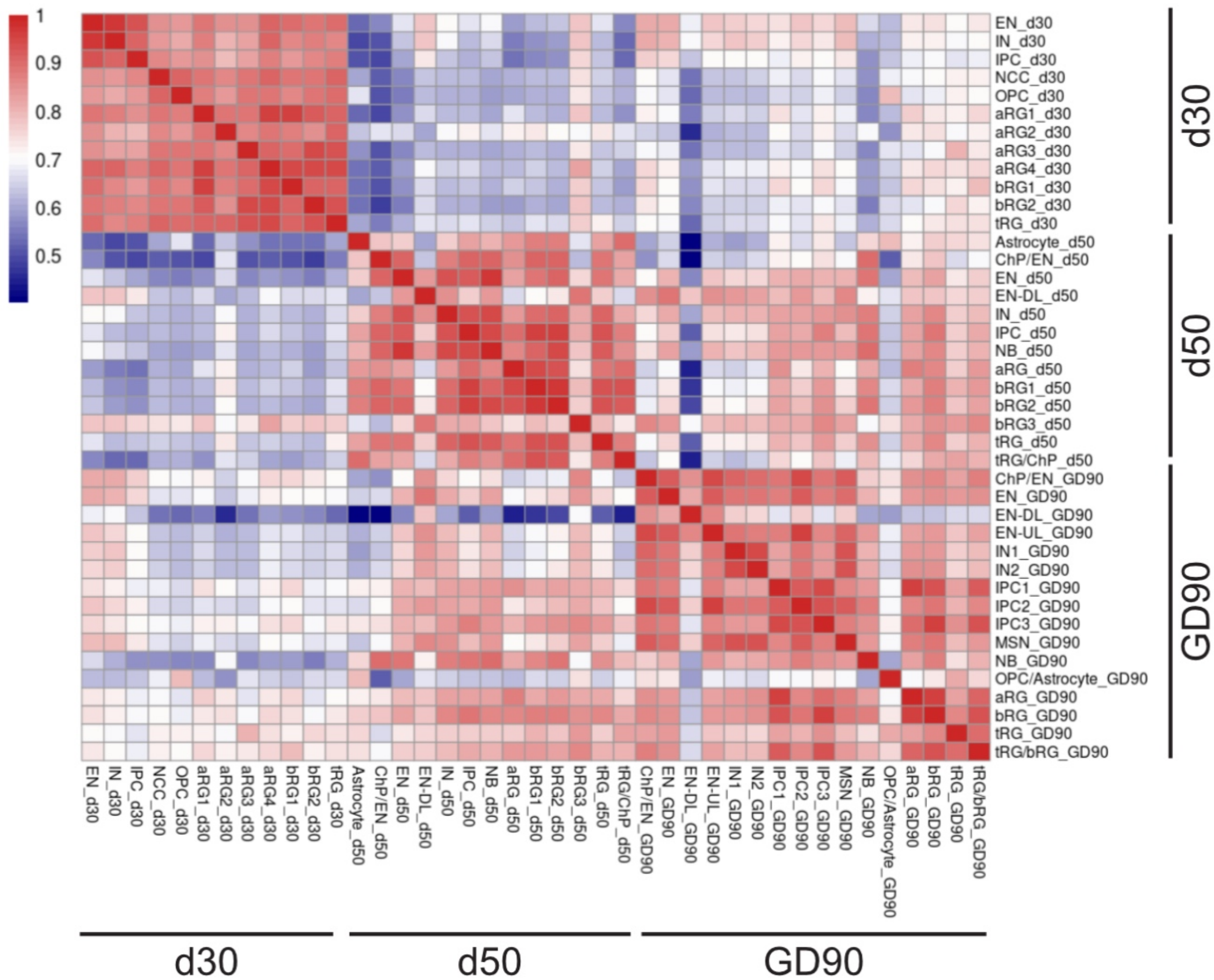

Fig. S9: The highest cross-sample correlations are observed between day 50 marmoset cerebral organoid clusters and GD90 fetal marmoset telencephalic clusters.

Heatmap of Pearson correlation matrix of scRNA-seq clusters for all marmoset scRNA-seq samples (day 30 and day 50 cerebral organoids and GD90 telencephalic tissue).

Table S1. (separate file)

Differentially expressed genes between day-3 and day-6-old human and marmoset embryoid bodies.

Table S2. (separate file)

Literature-derived markers used for cell type annotation of scRNA-seq data.

Table S3. (separate file)

Differentially expressed genes in day-30, day-40 and day-50-old marmoset FACS-enriched aRGs from cerebral organoids.

Table S4. Summary of the main findings

Main findings summarized according to the supporting data category (cerebral organoid data, *in vivo* data or published *in vivo* data).

Developmental aspect	Category	Cerebral organoid data (this study)	*In vivo* data (this study)	Published *in vivo* data
**Divergence in developmental timing**	Observation	Trajectory analysis indicates a shorter neurogenic path and an earlier gliogenic specification in marmoset compared to human.		Neurogenic length is shorter in marmoset than in human (36, 37).
	Potential mechanism	Marmoset and human aRG differ in transcriptional profiles and relative cluster composition.		
**VZ dominance**	Observation	VZ thickness at day 30 is comparable between species, but neuronal output is reduced in marmoset.		The VZ constitutes a larger proportion of germinal zones during much of neurogenesis (10)
	Potential mechanism	aRG in marmoset exhibit a longer cell cycle compared to human.		
		A population of EdU-negative aRG suggests the presence of quiescent or slowly cycling progenitors in marmoset organoids.		
**Transition VZ to SVZ dominance**	Observation	VZ thickness decreases markedly between day 30 and day 40, followed by reduced apical progenitor mitotic activity from day 40 to day 50.		The SVZ becomes the dominant germinal zone toward the end of neurogenesis (10).
	Potential mechanism	Expression of delamination-associated genes increases from day 30 to day 50.	Gene expression profiles from marmoset GD90 telencephalon correlate with day 50 organoid data.	
		The proportion of oblique and horizontal divisions increases between day 30 and day 50.		
		A relatively high proportion of non-vertical divisions is already present at day 30 (56.5%).		A relatively high proportion of oblique and horizontal divisions is already observed at GD60 (64.1%) (46).
		Tbr2$^+$ basal progenitors in the VZ and NeuN$^+$ neurons in basal regions are more abundant in marmoset compared to human at day 50.	Mean proportions of basal progenitors and neurons relative to total cells exceed those of apical progenitors.	
**bRG morphology**	Observation		bRG display relatively simple morphology.	
	Potential mechanism	PALMD expression is reduced in marmoset compared to human organoids.		
		PALMD levels are lower in bRG than in neurons and comparable to aRG.	PALMD levels are lower in bRG than in neurons and comparable to aRG.	

Table S5. (separate file)

Metadata for bulk RNA-seq and single-cell RNA-seq datasets used in this study.

Table S6. (separate file)

Neurodevelopmental genes used for comparison between FACS-enriched aRGs from marmoset cerebral organoids and GD90 aRGs and neurons from marmoset telencephalic tissue.

Table S7. (separate file)

Cell type-specific markers identified in this study.
